# Preparation of a ferulic acid–phospholipid complex to improve solubility, dissolution, and B16F10 cellular melanogenesis inhibition activity

**DOI:** 10.1186/s13065-017-0254-8

**Published:** 2017-03-22

**Authors:** Li Li, Yanhong Liu, Yan Xue, Jun Zhu, Xiaoyue Wang, Yinmao Dong

**Affiliations:** Beijing Key Laboratory of Plant Resources Research and Development, Beijing Technology and Business University, Haidianqufuchenglu 11hao dongqu8haolou 214shi, Beijing, 100048 People’s Republic of China

**Keywords:** Ferulic acid, Phospholipid, Solubility, Transdermal permeation, Melanin inhibition

## Abstract

**Background:**

We aimed to enhance the solubility, dissolution properties, and skin-whitening ability of ferulic acid (FA) by preparing a ferulic acid–phospholipid complex (FA–PC). The properties and melanogenesis inhibition activities of FA–PC were then elucidated.

**Methods:**

We characterized the complex via differential scanning calorimetry, Fourier transform infrared spectroscopy, scanning electron microscopy, solubility, and oil–water partition coefficient. A Strat-M^®^ membrane, a synthetic membrane possessing diffusion characteristics that are well-correlated with human skin, was used for the diffusion studies of FA–PC.

**Results:**

We found that the lipophilicity of FA improved when complexed with phospholipids, allowing FA–PC to release FA in a controlled pattern. In the same time, complexing with phospholipids also obviously enhanced inhibition of B16F10 cellular melanogenesis.

**Conclusions:**

FA–PC is a promising material for medicinal and cosmetic usages.

## Background

Ferulic acid (FA; 4-hydroxy-3-methoxycinnamic acid) is present in many foods, including wheat, rice, barley, oats, citrus fruits, and tomatoes [1]. FA has been shown to afford significant skin protection against UV-induced oxidative stress [2]. It reverts chronic UVB-induced oxidative damage in mice skin tumors by modulating the expression of vascular endothelial growth factor (VEGF), inducible nitric oxide synthase (iNOS), tumor necrosis factor (TNF)-*α*, and interleukin (IL)-6 [3]. It also modulates the expression of mutated *p53*, *Bcl*-*2*, and *Bax* in UVB-induced mice skin tumors [4]. Several studies have established that FA inhibits the expression of cytotoxic and inflammation-associated enzymes [5] and matrix metalloproteinases (MMPs), and attenuates the degradation of collagen fibers [6].

Phospholipid complexes are widely used in the pharmaceutical industry. They have good permeability and safety and are receiving increasing attention for application in cosmetics. Because phospholipids are biofunctional surfactants with good solubilizing properties, they can be used as carrier systems for less soluble drugs [7], improving transdermal permeation and cumulative penetration rate of topical drugs [8]. Transdermal permeation of drugs involves dissolution, distribution, and diffusion into the skin. Physical and chemical properties, especially the oil–water partition coefficient of the drug to be administered, affect this process [9].

Unfortunately, FA is a poorly soluble compound. We attempted to improve its solubility, skin penetration properties, and ability to inhibit melanogenesis by creating a novel ferulic acid–phospholipid complex (FA–PC) via a solvent evaporation method. The prepared FA–PC was then evaluated for various physical–chemical parameters. Differential scanning calorimetry (DSC; used to measure thermal behavior), Fourier transforms infrared spectroscopy (FTIR), and scanning electron microscopy (SEM), were utilized. Solubilities were measured and oil–water partition coefficients were calculated. In addition, a Strat-M^®^ membrane was used to evaluate skin permeability, and the ability for FA–PC to inhibit B16F10 cellular melanogenesis was investigated.

## Methods

### Materials

Powdered FA and arbutin (purity >99%) were purchased from Beijing HWRK Chem Co., Ltd. Soy lecithin (phosphatidylcholine, PC; purity >98%) was purchased from Shanghai Taiwei Co., Ltd. A Strat-M^®^ membrane was purchased from Merck Millipore (Darmstadt, Germany). Other chemical reagents were of analytical grade. Physical mixture (PM) was prepared by putting equimolar amount of ferulic acid and phospholipids into mortar and grinding the mixed material sufficiently.

### Cell culture

Mouse melanoma B16F10 cells were purchased from the Cell Bank of the Shanghai Institutes for Biological Sciences, Chinese Academy of Sciences. Cells were cultured in Dulbecco’s modified Eagle medium (DMEM) that was supplemented with 10% fetal bovine serum (BioWhittaker, Walkersville, MD, USA) and 1% penicillin–streptomycin (Gibco BRL, NY, USA). The cultures were incubated at 37 °C in a humidified atmosphere containing 5% CO_2_.

### Preparation of FA–PC using solvent evaporation

#### Screening for optimal proportion of ferulic acid and phospholipids

FA and phospholipids at molar ratios of 2:1, 1:1, 1:2, 1:3, and 1:4 were added to 100 mL round-bottom flasks and dissolved in anhydrous ethanol (FA, 2.0 mg/mL). The mixtures were stirred constantly at 40 °C for 1 h, and then the anhydrous ethanol was removed by rotary evaporation. The dried FA–PC complexes were placed in a desiccator for 24 h.

To determine the optimal ratio of FA to phospholipid, we measured the complexing rate of FA by UV spectrophotometry (UV-3150; Shimadzu, Japan). Briefly, the absorbance of prepared FA–PC samples in ethanol was determined at 323 nm. An equal amount of phospholipids dissolved in ethanol was used as a control, and a standard curve was constructed using FA. The complexing rate was expressed as mg of FA equivalents per g of dry weight.

#### Screening for optimal reaction temperature for FA–PC preparation

FA and phospholipids at a molar ratio of 1:1 were added to 100 mL round-bottom flasks and dissolved in anhydrous ethanol (FA, 2.0 mg/mL). The mixtures were stirred constantly at 20, 40, 60, or 80 °C for 1 h and then dried by rotary evaporation at 40 °C. Then, they were placed in desiccators in preparation for determination of FA content.

#### Screening for optimal reaction time for FA–PC preparation

FA and phospholipids at a molar ratio of 1:1 were added to a 100 mL round-bottom flask and dissolved in anhydrous ethanol (FA, 2.0 mg/mL). The mixtures were constantly stirred constantly at 40 °C for 15, 30 min, 1, 2, 3, or 4 h and then dried by rotary evaporation at 40 °C. Afterward, they were placed in desiccators in preparation for determination of FA content.

#### Screening for optimal FA concentration

FA and phospholipids at a molar ratio of 1:1 were added to 100 mL round-bottom flasks. Different volumes of anhydrous ethanol were added to each flask to yield FA concentrations of 1.0, 2.0, 4.0, 6.0, and 10 mg/mL. The mixtures were constantly stirred under 40 °C for 1 h and then dried by rotary evaporation at 40 °C. Afterward, they were placed in desiccators in preparation for determination of FA content.

### Characterization of FA–PC

#### Differential scanning calorimetry (DSC)

DSC was carried out with a differential scanning calorimeter (Q2000; TA Instruments, USA). FA, phospholipids (PC), a physical mixture of FA and phospholipids (PM), and FA–PC, were separately loaded onto aluminum pans and heated at a rate of 10 °C/min from 25 to 300 °C under a nitrogen atmosphere for thermal analysis.

#### Fourier transform infrared spectroscopy (FTIR)

The infrared spectra of FA, phospholipids, PM, and FA–PC were obtained via a liquid membrane method using an FTIR spectrometer (8200, Shimadzu, Japan). The spectra were recorded at a range of 400–4000 cm^−1^.

#### Scanning electron microscopy (SEM)

The morphologies of FA, PC, PM, and FA–PC were examined under scanning electron microscope (PhenomProX; Phenom World, Netherlands) at an acceleration voltage of 10 kV. Samples were sputter-coated with gold–palladium and observed at different magnifications.

### Solubility and oil–water partition coefficient

#### Solubility

The solubilities of powdered FA and FA–PC were determined by adding an excess of samples to 10.0 mL [10] of water or *n*-octanol and then shaking on a swing bed for 3 h at 37 °C. The mixtures were centrifuged at 15,000 rpm for 10 min to remove insoluble FA. Then, the supernatants were filtered through 0.45 µm membranes. Afterward, the filtrates were diluted tenfold with methanol and the FA content determined using a UV spectrophotometer (UV-3150; Shimadzu; Japan).

#### Oil–water partition coefficient

Samples (10 mL) of FA and FA–PC in water-saturated *n*-octanol were prepared and shaken. *n*-octanol–saturated water (10 mL) was added to each sample, and the miscible liquid was agitated for 24 h. Afterward, the samples were allowed to stand for layering. The FA concentration in each phase was determined by UV spectrophotometry (UV-3150; Shimadzu; Japan). Analyses were carried out in triplicate.

#### In vitro diffusion

In vitro diffusion studies were performed utilizing Franz diffusion cells (TK-20A; Shanghai Xie Kai Financial Information Service Co., Ltd.; China). In addition, we used Strat-M^®^ membranes, which are synthetic membranes with diffusion characteristics that correlate more closely to human skin than do animal skin models [11]. The membranes were clamped between the donor and receiver chambers of the vertical diffusion cells, and the receiver chambers were filled with phosphate-buffered saline (PBS; pH 7.4) to solubilize FA or FA–PC and ensure sink conditions.

The receiver chambers were kept at 37 °C using a thermostatic water bath, and the solutions inside the receiver chambers were magnetically stirred at 500 rpm throughout the experiment. About 3.0 mg of FA or FA–PC was placed in the donor chambers. At 1, 2, 4, 8, 16, and 24 h, the solutions (0.6 mL) inside the receiver chambers were removed and filtered through 0.45 μm membrane filters. The concentration of FA in each sample was determined using a validated high-performance liquid chromatography (HPLC) method.

Chromatographic separation was carried out using an Agilent 1260LC series system (Agilent Technologies, Palo Alto, CA, USA) equipped with an online vacuum degasser, quaternary pump, autosampler, thermostatted column compartment, and diode array detection (DAD). Agilent Technologies ChemStation software for liquid chromatography (LC; B.02.01) was used, and HPLC separation was performed using an Eclipse plus-C18 column (4.6 × 250 mm, 5 μm). The detection wavelength was set to 322 nm. The mobile phase consisted of water with 0.05% acetic acid (A) and methanol (B) (40:60, v/v). The flow rate was 1.0 mL/min. The column temperature was set at 30 °C. Cumulative corrections were made to ascertain the amount of FA released at each time interval. All measurements were performed in triplicate, and the percent of cumulative FA that permeated through the membrane (%Q) was plotted as a function of time.

### Inhibition of melanogenesis

#### B16F10 cell viability assay

Cell viability and cell proliferation were evaluated using a 3-(4,5-dimethylthiazol-2-yl)-2,5-diphenyltetrazolium bromide (MTT) assay [2]. B16F10 cells were pretreated with 0.25, 0.5, 1.0, 2.0, 4.0 mg/mL concentrations of FA and FA–PC. After incubation for 48 h, MTT solution (final concentration: 5 mg/mL) was added, and the cells were incubated for 3 h at 37 °C. Finally, the absorbance of each sample was measured on a microplate reader at 570 nm to obtain the percentage of viable cells.

#### Measurement of melanin content

Melanin content was measured as described previously [6] with some modifications. B16F10 melanoma cells were seeded (2 × 10^5^ cells/well in 3 mL of medium) in six-well culture plates and incubated overnight to allow cells to adhere. At the end of the treatment, the cells were washed with PBS and lysed with 1 M NaOH containing 10% dimethyl sulfoxide (DMSO) for 30 min at 80 °C. The absorbance (optical density; OD) was measured at 475 nm using a microplate reader. Melanin content was calculated using the following formula:

$${\text{Melanin}} \;{\text{content}} \;(\% ) = \frac{{{\text{OD}}475_{\text{sample}} }}{{{\text{OD}}475_{{{\text{blank}}\,{\text{control}}}} }} \times 100$$

### Data analysis

The statistical significance of the differences between the mean measurements of each treated group and that of the control group were determined using Dunnett’s t test. *P* values <0.05 were considered statistically significant.

## Results and discussion

### Optimal preparation of FA–PC

We determined that FA–PC was best prepared using an FA to phospholipid molar ratio of 1:1 according to the complexing rate. In addition, the optimal method required constant stirring of FA 6.0 mg/mL and phospholipids in anhydrous ethanol under 40 °C for 15 min. Anhydrous ethanol was removed after formation of FA–PC by using a rotary evaporator set at 40 °C, after which the dried residue was placed in a desiccator for 24 h. The resultant FA–PC was transferred to a glass bottle, flushed with nitrogen, and stored at room temperature.

### Solubility and oil–water partition coefficient of FA–PC

Table 1 shows the solubility of FA, PM, and FA–PC in water and in *n*-octanol. FA–PC exhibited good solubility in water and *n*-octanol (1.68 and 7.77 mg/mL, respectively), and the solubility of FA–PC in *n*-octanol was found to be significantly higher than that of FA (1.34 mg/mL).

**Table 1:** The solubility of FA, PM and FA–PC in water and n-octanol

Samples	Solubility (mg/mL)	
	Water	*n*-octanol
FA	0.67 ± 0.02	1.34 ± 0.03
PM	0.75 ± 0.05*	1.85 ± 0.02**
FA–PC	1.68 ± 0.01**	7.77 ± 0.06**

Data are presented as mean ± SEM of three independent experiments

* *P* <0.05, ** *P* <0.01, compared with FA

Lipophilicity is usually measured as a partition coefficient (*P*) between two immiscible phases. It is typically expressed as log *P*. The apparent partition coefficients of FA and FA–PC in the *n*-octanol/water system were determined. Results indicated that log *P* was higher for FA–PC (1.21) than for FA (0.99) when measured at pH 5.0. The slightly increased log *P* was related to the significantly improved *n*-octanol solubility of FA–PC compared to that of FA. The increased solubility of FA–PC in *n*-octanol may be explained by the amorphous characteristics of the FA–PC. As lipophilicity and permeability are well-correlated, these results suggest that the transdermal permeability of FA might be improved by applying it as a phospholipid complex.

### DSC

**Fig. 1:**
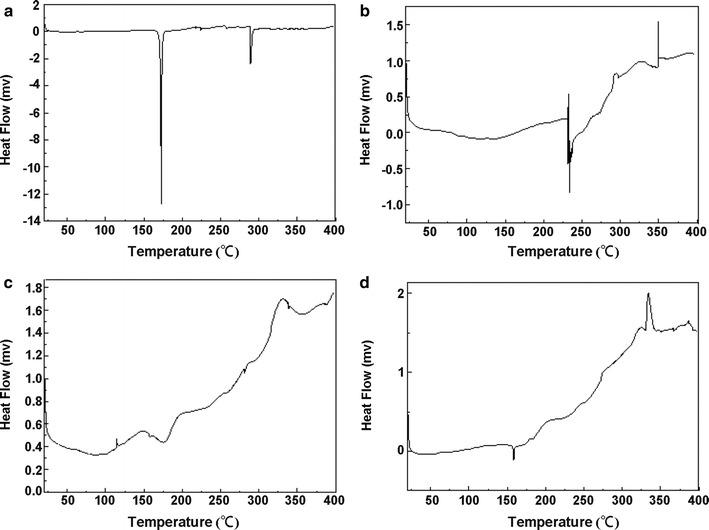
DSC thermograms of **a** FA, **b** PC, **c** PM, and **d** FA–PC

DSC is a reliable method for screening drug-excipient compatibility and provides maximum information about possible interactions between the drugs and excipients [10]. The presence of an interaction can be concluded from elimination of endothermic peaks, appearance of new peaks, change in peak shape and onset, peak temperature/melting point, and relative area or enthalpy. Figure 1 shows the DSC thermograms of FA (Fig. 1a), PC (Fig. 1b), PM (Fig. 1c), and FA–PC (Fig. 1d). The thermal curve for pure FA has a typical sharp endothermic melting at about 172.7 °C, indicative of its anhydrous and crystalline state, while that of phospholipids exhibit a minor endothermal peak at 231.7–248.6 °C. The DSC curve for PM, consisting of the superimposed thermal profiles for FA and phospholipids, shows no significant changes except for a small shift to a higher temperature (175.9 °C), indicating no interactions between the components. FA–PC has one major peak at 158.2 °C, which differs from the FA peak, indicating an interaction between FA and PC. Our results suggest that different degrees of interaction and/or amorphization in different mixtures or complexes can be obtained depending on their preparation method and this is associated with the differences in solubility.

### FTIR

**Fig. 2:**
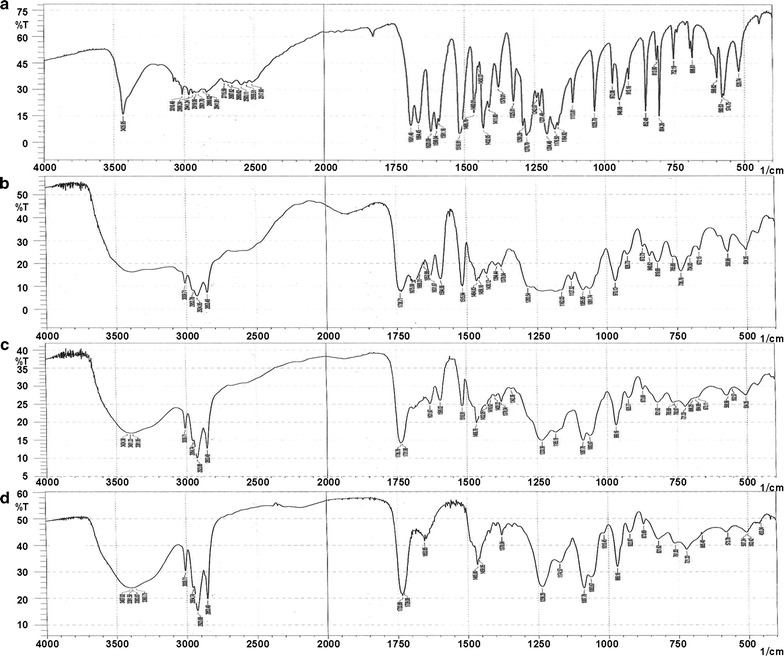
FTIR spectra of **a** FA, **b** FA–PC, **c** PM, and **d** PC

FTIR spectroscopy can confirm the formation of FA–PC by comparing the FA–PC spectrum with that of pure FA. Figure 2 shows the FTIR spectra of FA, PC, PM, and FA–PC. The FA spectrum (Fig. 2a) shows a characteristic hydroxyl stretching band at 3436 cm^−1^. It all becomes a wide singlet in the spectrums FA–PC, PM, phospholipid (Fig. 2b–d). The FA spectrum (Fig. 2a) exhibits characteristic peaks at 1620 cm^−1^ (C=C stretching) and 1450 cm^−1^ (C=C aromatic ring stretching). In the FA–PC spectrum (Fig. 2b), these two peaks are not visible, probably owing to weakening or removal or shielding by the phospholipid molecule.

The FA spectrum (Fig. 2a) exhibits characteristic unsaturated carboxyl peaks at 1691 cm^−1^ (C=O stretching), and 1664 cm^−1^ (C=C stretching). In the FA–PC spectrum (Fig. 2b), these two peaks are not visible, probably owing to the attractive forces between the negative carboxyl charge in FA and positive nitrogen charge in phospholipids. The phospholipid spectrum (Fig. 2d) has peaks at 1733 cm^−1^ (C=O stretching), 1238 cm^−1^ (P=O stretching), and 1087 cm^−1^ (P–O–C stretching). The peaks at 1733 and 1087 cm^−1^ are retained in the PM and FA–PC spectra, indicating non-involvement in formation of the complex. The phospholipid peak at 1283 cm^−1^ is not observable in the FA–PC spectrum probably because of the characteristic hydroxyl from FA being connected with P=O at 1283 cm^−1^ through van der Waals forces. The results indicate that FA was embedded into the ring structure composed of the negative phosphorus oxygen bond and positive nitrogen charge in phospholipids, which became the complex FA–PC.

### SEM

**Fig. 3:**
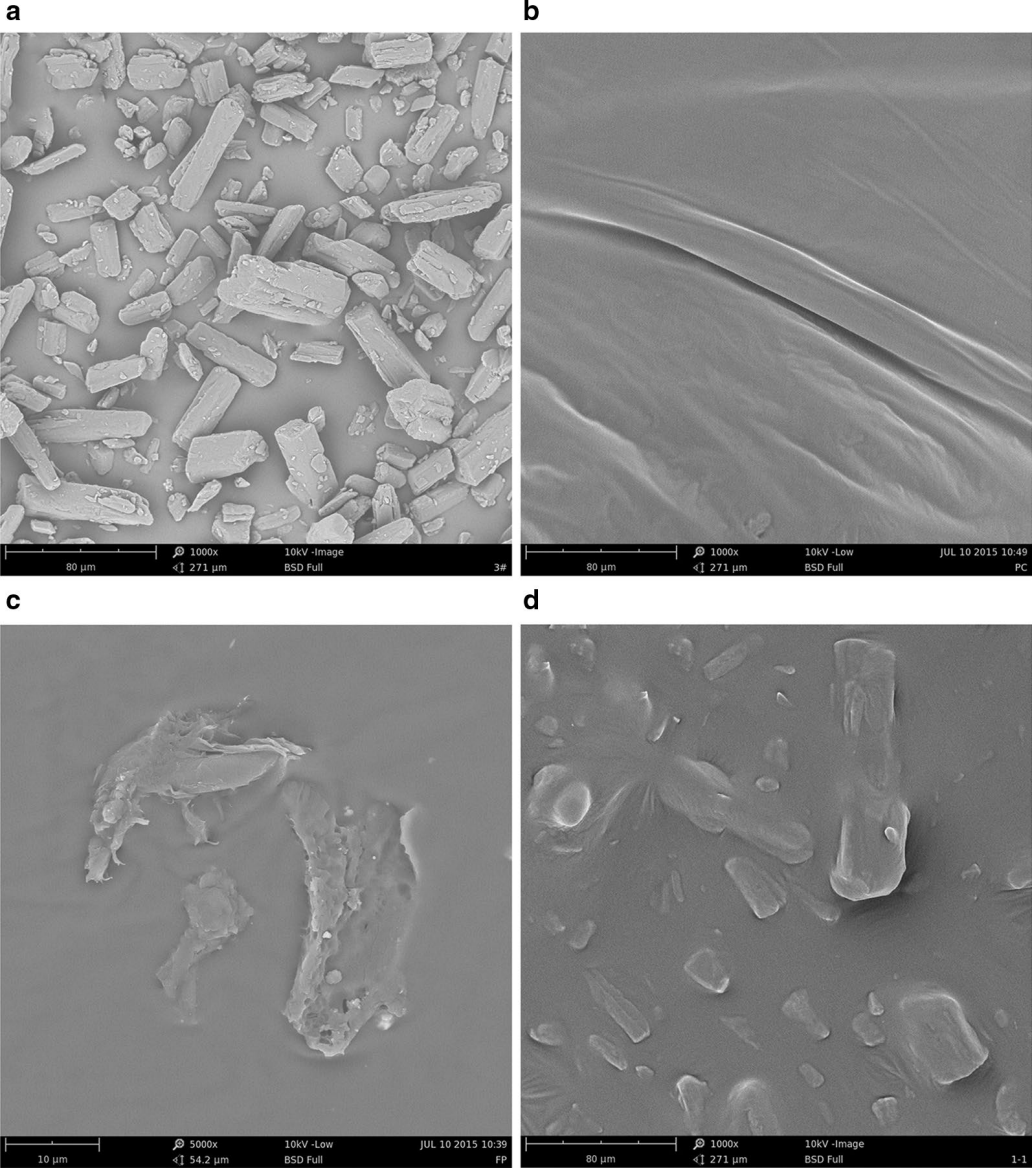
SEM images of **a** FA, **b** PC, **c** FA–PC, and **d** PM

The surface morphologies of FA, PC, PM, and FA–PC were investigated using SEM (Fig. 3). In Fig. 3c, FA appears crystalline, almost rectangular, while FA–PC particles (Fig. 3a) appear irregular in shape with a smooth surface. FA–PC possesses a significantly different shape and surface topography compared to that of FA and PC (Fig. 3b). This probably owes to the complete miscibility of FA in PC. In the PM scan (Fig. 3d), both FA and phospholipids are easily distinguishable.

### In vitro diffusion studies

Recently, the synthetic Strat-M^®^ membrane was introduced as a substitute for human skin in in vitro diffusion studies [11]. The Strat-M^®^ membrane is composed of two layers of polyethersulfone that are resistant to diffusion. The polyethersulfone layers lie atop one layer of polyolefin, which is more open and diffusive. This synthetic membrane is characterized by low batch-to-batch variability, thus providing more consistent data. Moreover, it has been shown that diffusion data for Strat-M^®^ membranes correlate well with those of human skin [11].

**Fig. 4:**
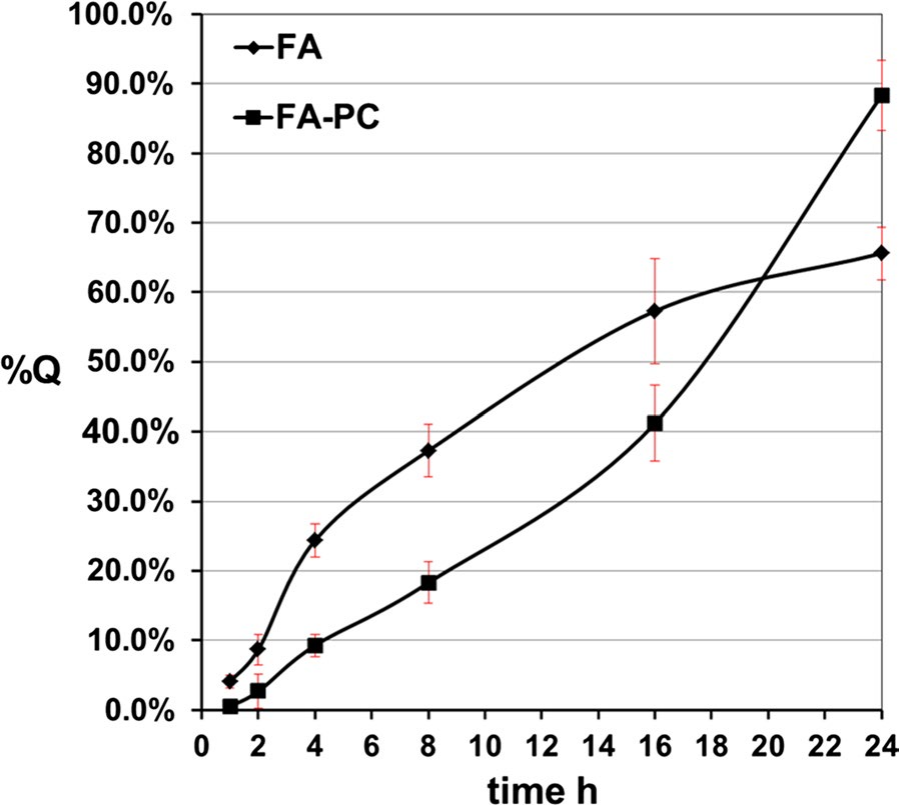
The % cumulative amount of FA and FA–PC that permeated through the membrane (%Q) plotted as a function of time

To evaluate the influence of PC on the in vitro diffusion properties of FA, the %Q of FA and FA–PC was plotted against time. The results in the present paper showed a trend that phospholipids significantly increased FA permeation into the Strat-M^®^ membrane. In addition, FA–PC resided longer on the Strat-M^®^ membrane than did FA (Fig. 4). Therefore, incorporation of phospholipids into FA maybe increases its residence time in stratum corneum and makes it more suitable for skin permeation. About the permeation time, although Strat-M^®^ membrane has been reported to have good correlation with those of human skin [11], it also has textural differences compared with human skin. The influence factors of the permeation times maybe included solvent, concentration of the compounds, pH value, et al. More experiments should be done in future analysis.

### Inhibition of melanogenesis

#### Effect of FA–PC on melanin synthesis

According to the literature, FA can inhibit cellular tyrosinase activity and melanogenesis in B16F10 melanoma cells through down regulation of the cellular proteins c-kit and ERK1/2 [12]. In the present study, FA–PC decreased melanin content in B16F10 cells more obviously than FA at tested concentrations of 0.25, 0.5, and 1.0 mg/mL (Table 2). Thus, we determined that FA–PC is more effective than FA at inhibiting melanin synthesis in B16F10 cells.

**Table 2:** Effects of FA and FA–PC cellular melanin content and cell viability in B16F10 melanoma cells

Samples	Concentration (mg/mL)	Cell viability (%)	Cellular melanin content (%)
Control	–	100.00 ± 7.26	100.0 ± 6.6
FA	0.25	104.19 ± 5.88	61.9 ± 8.4**
	0.5	83.31 ± 16.77	48.9 ± 8.0**
	1.0	77.99 ± 7.18	26.5 ± 0.0**
	2.0	12.13 ± 1.91	–
	4.0	11.10 ± 5.01	–
FA–PC	0.25	103.14 ± 7.54	32.4 ± 0.8**^##^
	0.5	110.95 ± 7.45	28.0 ± 2.0**^#^
	1.0	78.58 ± 9.99	30.3 ± 7.4**
	2.0	71.75 ± 10.12^##^	–
	4.0	44.27 ± 7.30^##^	–

Data are presented as mean ± SEM of three independent experiments. * *P* <0.05, ** *P* <0.01, compared with the control

Data are presented as mean ± SEM of three independent experiments. ^#^ *P* <0.05, ^##^ *P* <0.01, compared with FA

## Conclusion

We conclude that complexing FA with phospholipids improves FA’s water solubility, lipid solubility, bioavailability, and B16F10 cellular melanogenesis inhibition activity. The implications of our results include the potential use of FA–PC as a material in medicine or cosmetics.
